# Treatment of periprosthetic femoral fractures following total hip arthroplasty: results of an online survey of the European Hip Society

**DOI:** 10.1177/11207000211017115

**Published:** 2021-06-08

**Authors:** Martin Thaler, Carmen Weiss, Ricarda Lechner, Jean-Alain Epinette, Theofilos S Karachalios, Luigi Zagra

**Affiliations:** 1 Department of Orthopaedics and Traumatology, Medical University of Innsbruck, Innsbruck, Austria; 2 Orthopaedic Research and Documentation in Arthroplasty, Lille, France; 3 University General Hospital of Larissa, School of Health Sciences, Faculty of Medicine, University of Thessalia, Larissa, Thessalia, Greece; 4 IRCCS Istituto Ortopedico Galeazzi, Hip Department, Milan, Italy

**Keywords:** Classification, European Hip Society, periprosthetic fracture, surgery, total hip arthroplasty, Vancouver classification

## Abstract

**Background:**

Periprosthetic femoral fractures (PPF) are a devastating complication after total hip arthroplasty (THA). Both trauma and adult reconstruction surgeons or combined teams treat these fractures following management algorithms. The aim of this study is to investigate the current treatment of PPF by members of the European Hip Society (EHS).

**Methods:**

An online survey of the members of the European Hip Society (EHS) was conducted. 20 cases of periprosthetic fracture were presented and surgeons were asked to answer questions regarding classification, treatment and postoperative treatment protocol.

**Results:**

A total of 132 (130 male; 2 female) EHS members responded. Mean years in surgical practice was 18.8 (min. 1 year; max. 50 years). The preferred surgical method was combined open reduction and internal fixation (ORIF) (30.3%) for AG fractures, ORIF with cables (30.4%) for AL fractures, combined ORIF (cable and plate) for B1 fractures (49.2%), stem revision with cables for B2 fractures (73.1%), stem revision with cables for B3 (55.9%) fractures and combined ORIF (cable and plate: 55.5%) for C fractures. Surprisingly, 10.8% suggested various stem revision techniques for B1 and 17.4% for C fractures. Strong variations were observed regarding postoperative weight-bearing protocol.

**Conclusions:**

A strong consensus was found for the choice of conservative or surgical treatment of the different PPF types according to the Vancouver Classification. Various stem revision techniques were the preferred surgical techniques for Vancouver B2 (91.2%) and B3 (88.6%) fractures. However, for postoperative weight-bearing, when the ORIF technique was used, a significant variation of protocols was found.

## Introduction

Accompanying the increase in demand for primary total hip arthroplasties (THAs), an increase in complications such as periprosthetic femoral fractures (PPF) is expected.^1^ It has been postulated that the incidence of postoperative periprosthetic femoral fractures is approximately 0.1–2.1% for primary THA and around 3.6–20.9% for revision THA.^2–4^ According to the Australian National Joint Replacement Registry periprosthetic fracture is the most common reason for revision in patients aged over 75 years, and the risk is 3 times greater for cementless prostheses.^5^ Surgery for PPF with revision of the femoral component can be exceedingly demanding in terms of technique and is associated with a high rate of complications (18%) and re-operation (23%).^6^

The diagnosis, the management algorithm and the postoperative protocol regarding the treatment of PPF are complex. Various factors have an influence on these variables. The major ones are the location of the fracture relative to the implant, the state of fixation of the implant, and the influence of the quality of the surrounding bone. These 3 variables are described in the Vancouver Classification and form the basis for the established algorithm of management.^7^ Another variable is the surgeon’s training and experience. Both trauma and adult reconstruction surgeons or combined teams, where available, currently treat these patients without uniform management strategies.

We report on the results of an online survey designed to investigate the current practices of classification, management and postoperative rehabilitation protocols of PPF by experienced European surgeons who mainly practise adult reconstruction surgery. Questions on classification, indications for further modern imaging, and postoperative weight-bearing protocols were included and, if operative treatment was recommended, details of surgical technique were also recorded.

## Materials

For this cross-sectional study, an online survey was developed. This survey was then sent to all 510 members of the European Hip Society (EHS). The study has been approved by the local ethics committee.

The survey consisted of 7 personal demographic questions and 20 PPF example cases (single answer). The cases provide patient characteristics and anteroposterior (AP) as well as lateral fracture radiographs and ask surgeons to decide on treatment (conservative vs. surgery), and whether additional investigations (computed tomography (CT), inflammation parameters, axial and lateral x-rays) should be carried out. If the respondent decided to perform surgery, he or she was asked to define his or her surgical technique: open reduction and internal fixation (ORIF) with a plate alone, ORIF with cables alone, combined ORIF with a cable and a plate, stem revision with a plate, stem revision with cables or other. If the respondent opted to revise the stem, he or she was asked to state the fixation method (cemented versus non-cemented) for the revision stem. The participant was also asked to give his recommendations regarding weight-bearing restrictions after surgery. Each participant was asked to complete all case-relevant questions for every example case. For this reason, the number of responses exceeds the total number of respondents.

All questions included in the survey are shown in Table 1. Some examples of the radiographs are shown in the Appendix. Data were collected using SurveyMonkey (http://www.surveymonkey.com): an online data collection program.

**Table 1.** Survey questions and answers.

Question	Answer
Gender	Female
	Male
In what country do you practise?	
What are you specialized in?	Hip arthroplasty
	Knee arthroplasty
	Trauma
	General orthopedics
	Shoulder and elbow
	Hand and wrist
	Foot and ankle
	Spine
	Sports medicine
	Pediatrics
	Conservative hip surgery
	Other
How many arthroplasty surgeries do you perform per year?	
How many fractures do you operate on per year?	0–25
	25–50
	50–100
	100–200
	>200
How many surgeries for periprosthetic fractures do you perform per year?	0–10
	10–20
	20–50
	50–100
	>100
Questions per case example	
Please classify the periprosthetic fracture shown in the X-ray	Vancouver Type AG Vancouver Type AL
	Vancouver Type B1
	Vancouver Type B2
	Vancouver Type B3
	Vancouver Type C
Given the information presented here (X-ray + medical record), would you treat this patient?	Surgically
	Conservatively
Would you need more investigations in order to choose your treatment strategy	No
	Yes
If you opted for surgical treatment, please state what kind of surgery you would perform:	Osteosynthesis with a plate alone
	Osteosynthesis with cables alone
	Combined osteosynthesis with a cable and a plate
	Stem revision with a plate
	Stem revision with cables
	Other
If you opted for stem revision, what kind of stem fixation would you perform?	Cemented
	Non-cemented
Would you recommend weight-bearing restrictions after surgery?	No weight bearing for 6 weeks
	Partial weight bearing for 6 weeks
	No weight bearing for 12 weeks
	Partial weight bearing for 12 weeks
	Full weight bearing
	Other

All data from the online database were stated as frequencies and percentages.

## Results

A total of 132 (130 male; 2 female) EHS members responded. Mean years in surgical practice was 18.8 (min.1 year; max. 50 years). Frequencies of PPF, THAs and fractures in general treated per year per surgeon are summarised in Tables 2–4.

**Table 2.** Number of treated periprosthetic fractures (PPF) per year.

Periprosthetic fractures performed per year		
0–10	67	50.8%
10–20	48	36.3%
20–50	15	11.4%
50–100	2	1.5%
Total	132	100%

**Table 3.** Number of total hip arthroplasty (THA) surgeries performed per year.

THAs performed per year		
0–25	8	6%
25–50	14	10.6%
50–100	26	19.8%
100–200	46	34.8%
>200	38	28.8%
Total	132	100%

**Table 4.** Number of treated fractures per year.

Fractures operated per year		
0–25	57	43.2%
25–50	34	25.8%
50–100	23	17.4%
100–200	11	83%
>200	7	53%
Total	132	100%

### Vancouver AG fractures

The majority of participants (69%) did not request further investigations for their treatment decision, while 24% would want a CT scan and 6% a lateral x-ray.

If a fracture was classified as Vancouver AG, 62.5% of the respondents recommended surgery and 37.5% conservative treatment. Of the respondents who decided to perform surgery, 39.2% recommended ORIF with a plate, while 33% recommended combined ORIF (Table 5).

**Table 5.** Surgical treatment recommendations according to the Vancouver Classification: % (*n*) for all example cases.

Vancouver type						
	AG	AL	B1	B2	B3	C
Surgical technique						
Combined ORIF (cable and plate)	33% (50)	15.2% (9)	49.5% (165)	5.1% (52)	4.2% (15)	55.5% (93)
ORIF with cables	24.3% (37)	30.4% (19)	28.7% (96)	2.5% (26)	0.9% (3)	0.6% (1)
ORIF with plate	39.2% (61)	2.2% (1)	11.5% (38)	0.4% (4)	0.6% (2)	26.5% (44)
Stem revision and cable and plate	1.4% (2)		3.1% (10)	0.8% (8)	2.1% (7)	
Stem revision and plate	0.7% (1)	6.5% (4)	1.6% (5)	17.3% (178)	30.6% (106)	5.2% (9)
Stem revision and cables	1.4% (2)	45.7% (28)	5.3% (18)	73.1% (751)	55.9% (194)	11% (18)
Cables and allograft strut			0.3% (1)			
Distal locking stem			0.8% (8)	0.8 (8)	1.8% (6)	1.2% (2)
Proximal femur replacement					3% (10)	
Allograft prosthesis composite					0.9 % (3)	

### Vancouver AL fractures

Of the participants 59.7% reported that they do not need further investigations, while 32.4% would ask for a CT scan and 7.9% for lateral x-rays of the fracture. In the case of Vancouver AL fractures, 68.7% of the respondents reported that they would recommend conservative treatment, while 31.3% stated that surgical treatment would be appropriate for these fractures. For surgical treatment of the AL fractures presented in the current survey, 45.7% stated that stem revision and cables would be indicated with an uncemented stem in 88% (Table 5).

### Vancouver B1 fractures

Most participants (53.9%) wanted no further investigations. However, 32.6% would request a CT scan of the fracture and 12.4% a lateral x-ray.

Strong agreement (72.3%) was found for surgical treatment of B1 fractures. Regarding the surgical technique, the majority stated that they would perform a combined ORIF (plate + cable: 49.5%), 28.7% reported that ORIF with cables would be their preferred choice, and 11.5% favored ORIF with a plate (Table 5). Various stem revision techniques were suggested by 10.8% of the surgeons. Uncemented stems were preferred by 85.3%.

### Vancouver B2 fractures

The vast majority of respondents (67.9%) stated that they would need no further investigations, while 20.6% would want a CT scan, 6.9% a lateral x-ray, 1.9% a lateral x-ray and a CT scan.

For Vancouver B2 fractures there was a strong consensus (98.2%) to treat patients operatively. Of the participants 73.1% would perform a stem revision with cables, while 17.3% preferred a stem revision with a plate, and 5.1% would perform a combined ORIF (Table 5). For stem revision 89.5% preferred uncemented stems.

### Vancouver B3 fractures

No further investigations were required by 60.9% of all participants. 27% wanted an additional CT scan of the fracture site, 2.7% a lateral x-ray, 2.1% a lateral x-ray and a CT scan.

Of the participants 98% would treat B3 fractures surgically. The preferred type of surgery was stem revision with cables (55.9%), or stem revision with plate (30.6%), while combined ORIF (cable and plate) was preferred by 4.2% of the respondents (Table 5).

### Vancouver C fractures

The majority (73.4%) of the respondents needed no further investigations. 19% stated that they would ask for a CT scan, while 5.3% wanted an x-ray of the whole femur including knee joint and 2.3% would request a lateral x-ray and a CT scan.

Strong consensus (98.2%) was observed for the indication for surgery. Regarding the surgical technique, 55.5% preferred a combined ORIF (cable and plate), 26.5% ORIF with a plate, 11% stem revision with cables and 5.2% stem revision in combination with a plate (Table 5). For stem revision 73.4% would opt for an uncemented stem.

### Weight-bearing

Recommendations regarding weight-bearing restrictions for all fractures are summarised in Table 6.

**Table 6.** Weight-bearing (WB) restrictions after surgery for all example cases according to the Vancouver classification.

	Full WB	Partial WB for 6 weeks	Partial WB for 12 weeks	No WB for 6 weeks	No WB for 12 weeks
Vancouver type					
AG % *n*	17 (27)	55.1 (86)	4.8 (7)	20.4 (32)	2.7 (4)
AL % *n*	13.3 (8)	55.6 (34)	11.1 (7)	20 (12)	0
B1 % *n*	49.1 (164)	32.5 (108)	6.3 (108)	9 (30)	3.1 (10)
B2 % *n*	18.7 (192)	60 (616)	11.9 (122)	7 (72)	2.4 (25)
B3 % *n*	35.5 (126)	28.6 (101)	17.1 (61)	12 (42)	6.8 (24)
C % *n*	4.7 (8)	34.7 (59)	26.9 (46)	20.7 (35)	13 (22)

## Discussion

As the number of THAs being performed worldwide continues to increase, so does the incidence of PPF. PPFs are a severe complication of hip arthroplasty. The choice of treatment modality depends on fracture, implant, and bone characteristics. For classification in our study, we used the Vancouver system, which is the most widely accepted classification scheme for grouping fractures with similar characteristics from which a treatment algorithm can be derived.^7,8^ Anatomic location partitions divide fractures into one of 3 categories with Type A occurring around the trochanteric region, Type B near or just distal to the femoral stem, and Type C well below the femoral stem. Type B fractures are subdivided based on stability and bone stock. B1 implies a well-fixed stem, B2 a loose stem with good bone stock, and B3 designates poor surrounding bone stock. Type AG fractures are located at the greater trochanter. Type AL fractures are located at the lesser trochanter.^8^ The Vancouver Classification shows good intra- and inter-observer variability.^9,10^ However, most publications studied classification and management of PPF in a small group of surgeons located in the same region.^11,12^ A cross-sectional overview of common clinical routine across Europe has not been investigated.

Vancouver type A fractures make up 2.8–4.7% of all PPF and consequently the treatment recommendations varied strongly.^13^ Our results show that the current practice of European surgeons is to treat the majority of AG (62.5%) and AL (68.5%) fractures conservatively. When surgery is performed for an AG fracture ORIF is the surgical method preferred by 96.5% of the participants. ORIF would be performed either with cable (24.3%), plate (39.2%), or a combination of both (33%). When surgery is performed for an AL fracture, stem revision was preferred by 52.2% of the respondents, and ORIF by 47.8%. The most preferred ORIF technique for AL fractures was ORIF with cables (30.4%).

The treatment of B1 fractures is very challenging, as it is well known that B1 fractures have a poorer outcome than B2 or B3 fractures.^14^ Various studies show that some B1 fractures were probably B2 fractures and were therefore judged to have a fixed component.^15^ This misclassification could contribute to the poorer outcome in this fracture group.^16,17^ Surprisingly, 27.7% of the respondents recommended conservative treatment for B1 fractures. For surgical treatment ORIF (89.7%) was the preferred choice. The majority of surgeons (49.5%) would perform ORIF with plate and cables. However, 10.8% of the respondents preferred various forms of stem revision techniques.

Nearly all (98%) respondents recommended surgical treatment for Vancouver B2, B3 and C PPF. 1 systematic review showed that as per consensus, most Vancouver B2 and B3 fractures reported in the literature are treated with revision THA with or without internal fixation.^15^ Our study results support this already established concept of treating Vancouver B2 and B3 fractures with stem revision.^15,18^ Of the EHS surgeons 91.2% treat B2 fractures and 94.3% treat B3 fractures with stem revision. One review also mentioned that open reduction with internal fixation (ORIF) might be a valuable option for the treatment of Vancouver B2 and B3 fractures. However, in this report, the authors also concluded that their review is limited due to the lack of data and methodological weaknesses in the literature supporting this concept.^19^ For B2 fractures only 8% and for B3 fractures only 5.7% of EHS members would perform ORIF. This is consistent with published data.^18^ ORIF for Vancouver B2 and B3 fractures might be limited to patients in palliative care and without great expectations. Stem revision with cables (73.1% B2 fractures; 55.9% B3 fractures) is the preferred surgical method according to our results. Strut allografts for B2 and B3 fractures are very rarely used. Our results show that for Vancouver C fractures ORIF (82.9%) is the preferred surgical option. However, 17.4% of the respondents preferred various types of stem revision techniques. Therefore, arthroplasty surgeons treat type C fractures in principle by means of ORIF.^20^

Despite the fact that published reports state that additional CT did not improve the evaluation of implant loosening, some participants would require a CT scan for classification of the PPF. CT scans are often performed when loosening cannot be clearly assessed from radiographs.^21^ In our study, a CT additional to the provided AP and lateral x-ray of the fracture would be requested by 24% of the respondents for AG fractures, by 32.4% for AL fractures, 32.6% for B1 PPF, 20.6% for B2 PPF, 27% for B3 fractures and by 19% for C fractures. CT scans in general increase the visibility and classification of a fracture, help define bone quality and osteolysis and give the surgeon more information before entering the operating room.

Little is known about weight-bearing recommendations after PPF. Full weight-bearing as soon as possible, together with full recovery of ambulatory status should be the aims of any surgery for PPF. However, our results show that partial weight-bearing for 6 weeks after surgery was recommended for AG PPF (55.1%), AL fractures (55.6%) and B2 fractures (60%). Although most B1 fractures are treated with ORIF, full weight-bearing would be allowed by 49.1% of the participants. For B2 fractures, partial weight-bearing for 6 weeks was predominantly recommended (Table 6). Of the participants 35.5% recommended full weight-bearing for B3 fractures, while 28.6% recommended partial weight-bearing for 6 weeks after PPF surgery. It may be assumed that B3 fractures mainly occur in older patients and partial weight-bearing is difficult to manage in these old patients; however, many respondents allow full weight-bearing after surgery for B3 fractures.

Our study has several limitations. All cases provided in this study had PPF of uncemented stems. For cemented stems, the use of the cement-in-cement technique for femoral component revisions has also been well described. This technique for the management of selected Vancouver B2 periprosthetic femur fractures offers another option for treating B2 fractures.^22^ However, due to the increasing number of cementless implants and the higher risk of fracture with this type of fixation it makes sense to analyse uncemented stems.^23^ This study was conducted on only a selection of international arthroplasty specialists. However, 30% of EHS members are professors and therefore we can presume that the EHS members participating in the current survey are international opinion leaders in the field of revision arthroplasty. In addition, the participating surgeons had a mean of 18.8 years in clinical practice. We thus expect these results from a very experienced group to be internationally representative. The complex nature of our study, because of the inclusion of multiple aspects of PPF management, may exert a limiting influence on interpretation. However, our study demonstrates trends and differences in the management of PPF in Europe. 27% of respondents recommended non-surgical treatment for B1 fractures, and 17.4% recommended revision surgery for type C fractures. Poor bone quality in type C fractures might have an impact on the surgeon’s decision to recommend a revision surgery. Regarding B1 fractures 40.2% of the surgeons would need a CT scan and 9.8% lateral x-rays to choose their treatment strategy. Additional imaging in these B1 fractures might lead to smaller variations regarding treatment recommendations.

The present study was designed to provide an overview of current practice in the treatment of PPF in Europe. To the best of our knowledge, this is the first study to explore PPF treatment strategies chosen by a European sample of experienced orthopaedic surgeons who mainly practise adult reconstruction surgery. Variations in practice could suggest the need for more standardised protocols for both diagnosis and management.

## Research Data

sj-jpg-1-hpi-10.1177_11207000211017115 – for Treatment of periprosthetic femoral fractures following total hip arthroplasty: results of an online survey of the European Hip Society Click here for additional data file. sj-jpg-1-hpi-10.1177_11207000211017115 for Treatment of periprosthetic femoral fractures following total hip arthroplasty: results of an online survey of the European Hip Society by Martin Thaler, Carmen Weiss, Ricarda Lechner, Jean-Alain Epinette, Theofilos S Karachalios and Luigi Zagra in HIP International This article is distributed under the terms of the Creative
Commons Attribution 4.0 License ( http://www.creativecommons.org/licenses/by/4.0/) which
permits any use, reproduction and distribution of the work without
further permission provided the original work is attributed as specified
on the SAGE and Open Access pages ( https://us.sagepub.com/en-us/nam/open-access-at-sage).

sj-jpg-2-hpi-10.1177_11207000211017115 – Supplemental material for Treatment of periprosthetic femoral fractures following total hip arthroplasty: results of an online survey of the European Hip Society Click here for additional data file. Supplemental material, sj-jpg-2-hpi-10.1177_11207000211017115 for Treatment of periprosthetic femoral fractures following total hip arthroplasty: results of an online survey of the European Hip Society by Martin Thaler, Carmen Weiss, Ricarda Lechner, Jean-Alain Epinette, Theofilos S Karachalios and Luigi Zagra in HIP International

sj-jpg-3-hpi-10.1177_11207000211017115 – Supplemental material for Treatment of periprosthetic femoral fractures following total hip arthroplasty: results of an online survey of the European Hip Society Click here for additional data file. Supplemental material, sj-jpg-3-hpi-10.1177_11207000211017115 for Treatment of periprosthetic femoral fractures following total hip arthroplasty: results of an online survey of the European Hip Society by Martin Thaler, Carmen Weiss, Ricarda Lechner, Jean-Alain Epinette, Theofilos S Karachalios and Luigi Zagra in HIP International

sj-jpg-4-hpi-10.1177_11207000211017115 – Supplemental material for Treatment of periprosthetic femoral fractures following total hip arthroplasty: results of an online survey of the European Hip Society Click here for additional data file. Supplemental material, sj-jpg-4-hpi-10.1177_11207000211017115 for Treatment of periprosthetic femoral fractures following total hip arthroplasty: results of an online survey of the European Hip Society by Martin Thaler, Carmen Weiss, Ricarda Lechner, Jean-Alain Epinette, Theofilos S Karachalios and Luigi Zagra in HIP International

sj-jpg-5-hpi-10.1177_11207000211017115 – Supplemental material for Treatment of periprosthetic femoral fractures following total hip arthroplasty: results of an online survey of the European Hip Society Click here for additional data file. Supplemental material, sj-jpg-5-hpi-10.1177_11207000211017115 for Treatment of periprosthetic femoral fractures following total hip arthroplasty: results of an online survey of the European Hip Society by Martin Thaler, Carmen Weiss, Ricarda Lechner, Jean-Alain Epinette, Theofilos S Karachalios and Luigi Zagra in HIP International

sj-jpg-6-hpi-10.1177_11207000211017115 – Supplemental material for Treatment of periprosthetic femoral fractures following total hip arthroplasty: results of an online survey of the European Hip Society Click here for additional data file. Supplemental material, sj-jpg-6-hpi-10.1177_11207000211017115 for Treatment of periprosthetic femoral fractures following total hip arthroplasty: results of an online survey of the European Hip Society by Martin Thaler, Carmen Weiss, Ricarda Lechner, Jean-Alain Epinette, Theofilos S Karachalios and Luigi Zagra in HIP International

sj-jpg-7-hpi-10.1177_11207000211017115 – Supplemental material for Treatment of periprosthetic femoral fractures following total hip arthroplasty: results of an online survey of the European Hip Society Click here for additional data file. Supplemental material, sj-jpg-7-hpi-10.1177_11207000211017115 for Treatment of periprosthetic femoral fractures following total hip arthroplasty: results of an online survey of the European Hip Society by Martin Thaler, Carmen Weiss, Ricarda Lechner, Jean-Alain Epinette, Theofilos S Karachalios and Luigi Zagra in HIP International

sj-jpg-8-hpi-10.1177_11207000211017115 – Supplemental material for Treatment of periprosthetic femoral fractures following total hip arthroplasty: results of an online survey of the European Hip Society Click here for additional data file. Supplemental material, sj-jpg-8-hpi-10.1177_11207000211017115 for Treatment of periprosthetic femoral fractures following total hip arthroplasty: results of an online survey of the European Hip Society by Martin Thaler, Carmen Weiss, Ricarda Lechner, Jean-Alain Epinette, Theofilos S Karachalios and Luigi Zagra in HIP International
