# Supplementary figures and images for: Treatment of periprosthetic femoral fractures following total hip arthroplasty: results of an online survey of the European Hip Society
Source: Hip Int. 2021 Jun 8;33(1):126–32. doi: 10.1177/11207000211017115 (PMC9827483; doi:10.1177/11207000211017115)

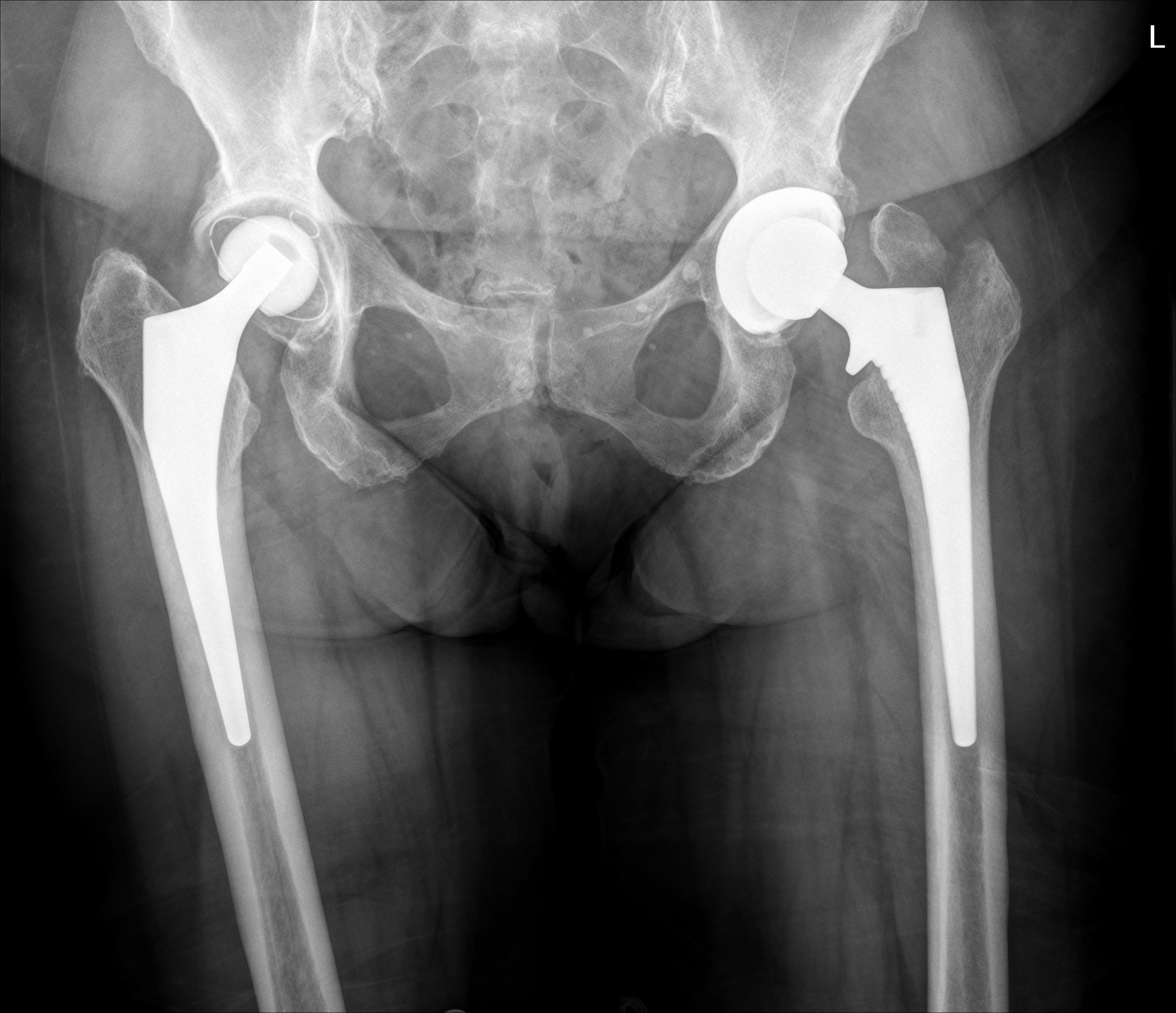

Supplement: sj-jpg-1-hpi-10.1177_11207000211017115 – for Treatment of periprosthetic femoral fractures following total hip arthroplasty: results of an online survey of the European Hip Society [file sj-jpg-1-hpi-10.1177_11207000211017115.jpg]

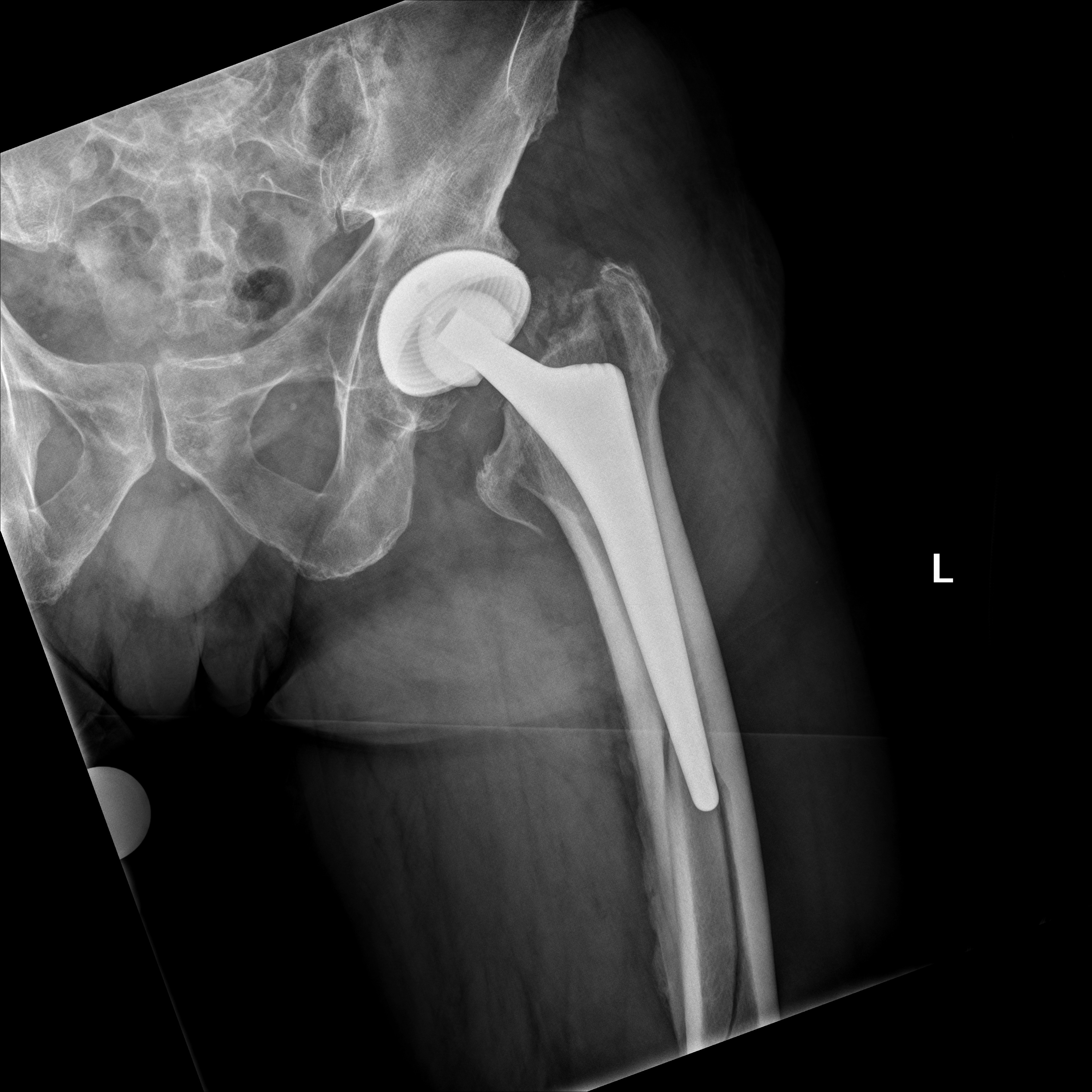

Supplement: sj-jpg-2-hpi-10.1177_11207000211017115 – Supplemental material for Treatment of periprosthetic femoral fractures following total hip arthroplasty: results of an online survey of the European Hip Society [file sj-jpg-2-hpi-10.1177_11207000211017115.jpg]

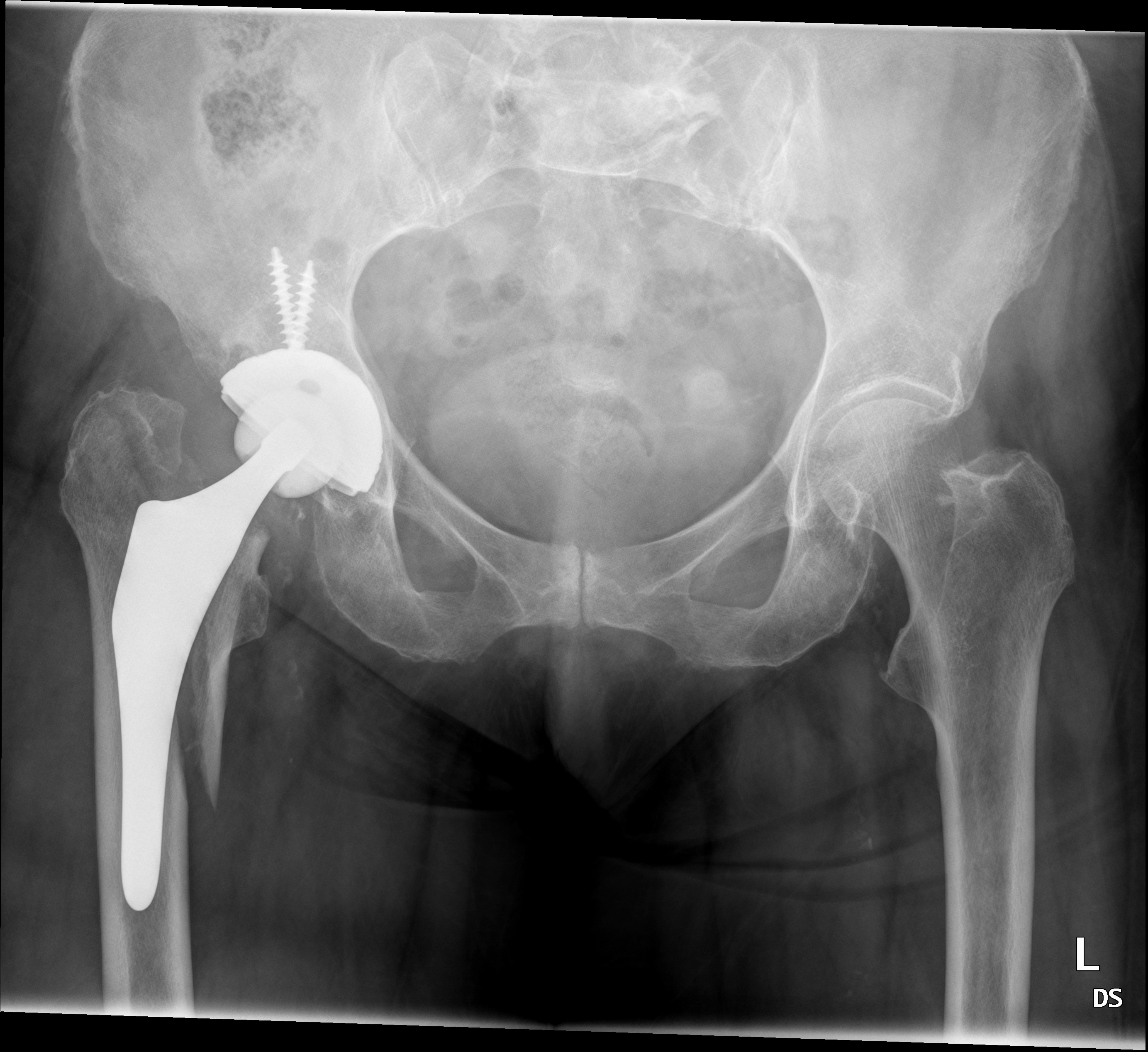

Supplement: sj-jpg-3-hpi-10.1177_11207000211017115 – Supplemental material for Treatment of periprosthetic femoral fractures following total hip arthroplasty: results of an online survey of the European Hip Society [file sj-jpg-3-hpi-10.1177_11207000211017115.jpg]

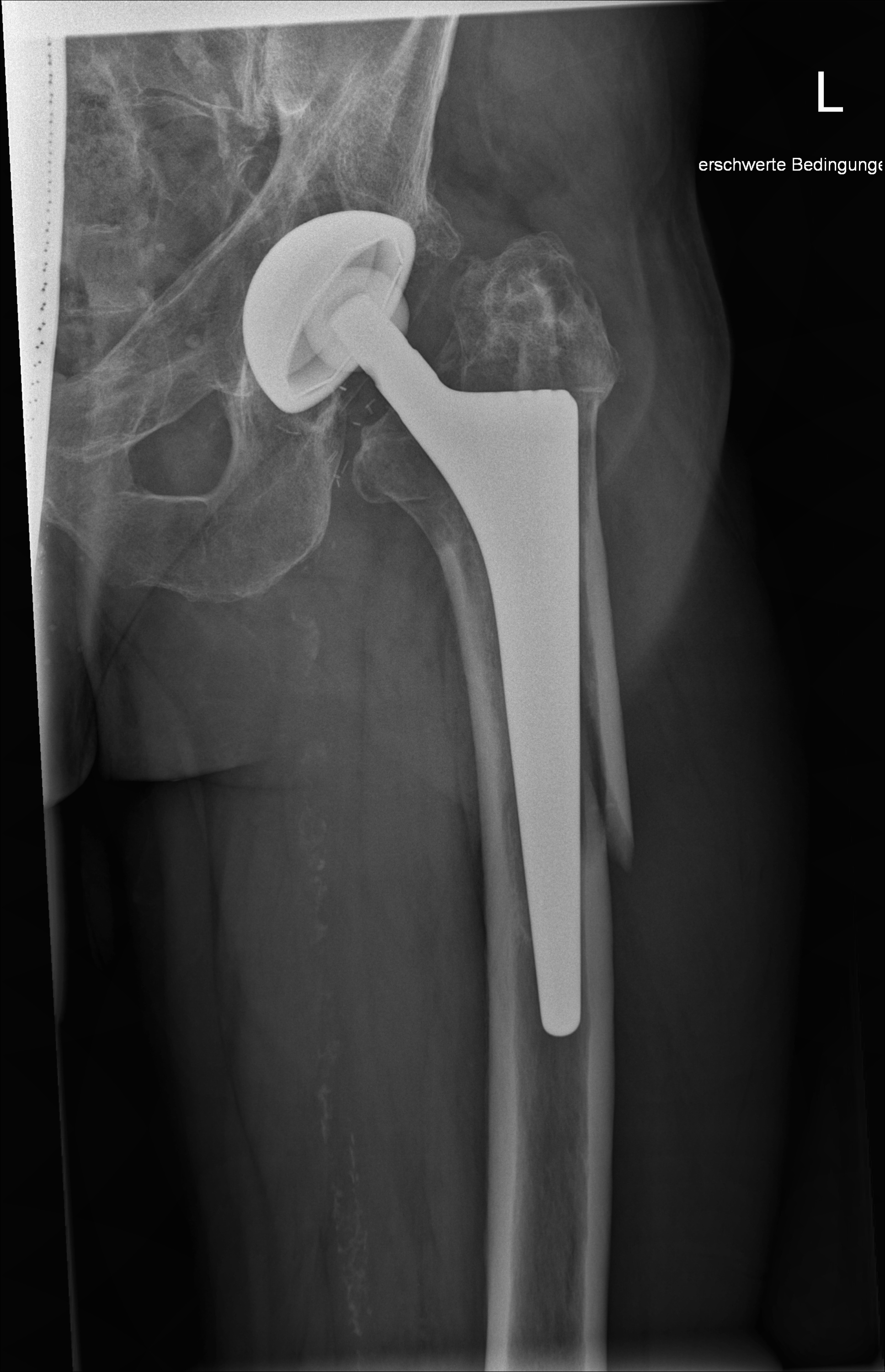

Supplement: sj-jpg-4-hpi-10.1177_11207000211017115 – Supplemental material for Treatment of periprosthetic femoral fractures following total hip arthroplasty: results of an online survey of the European Hip Society [file sj-jpg-4-hpi-10.1177_11207000211017115.jpg]

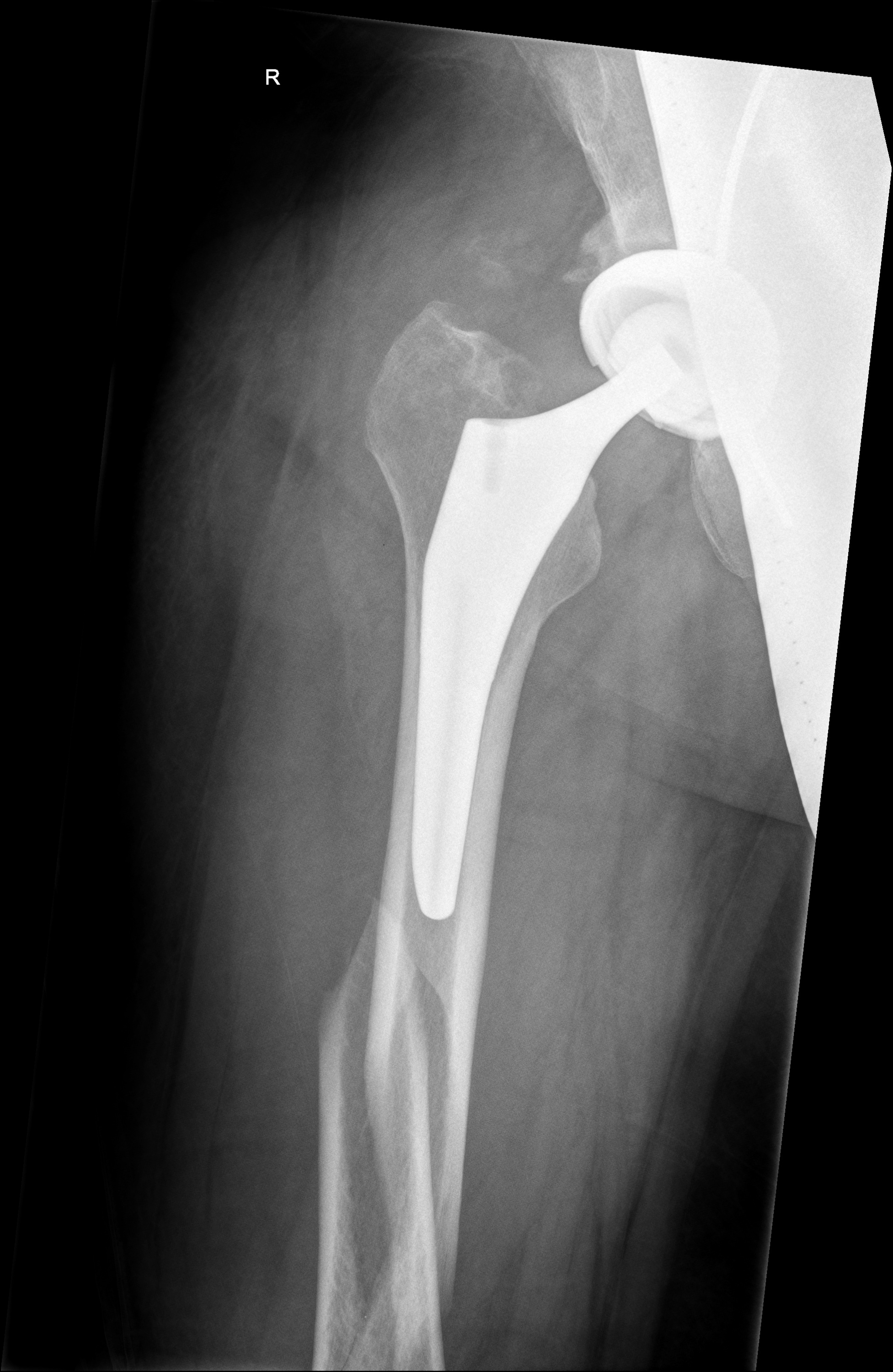

Supplement: sj-jpg-5-hpi-10.1177_11207000211017115 – Supplemental material for Treatment of periprosthetic femoral fractures following total hip arthroplasty: results of an online survey of the European Hip Society [file sj-jpg-5-hpi-10.1177_11207000211017115.jpg]

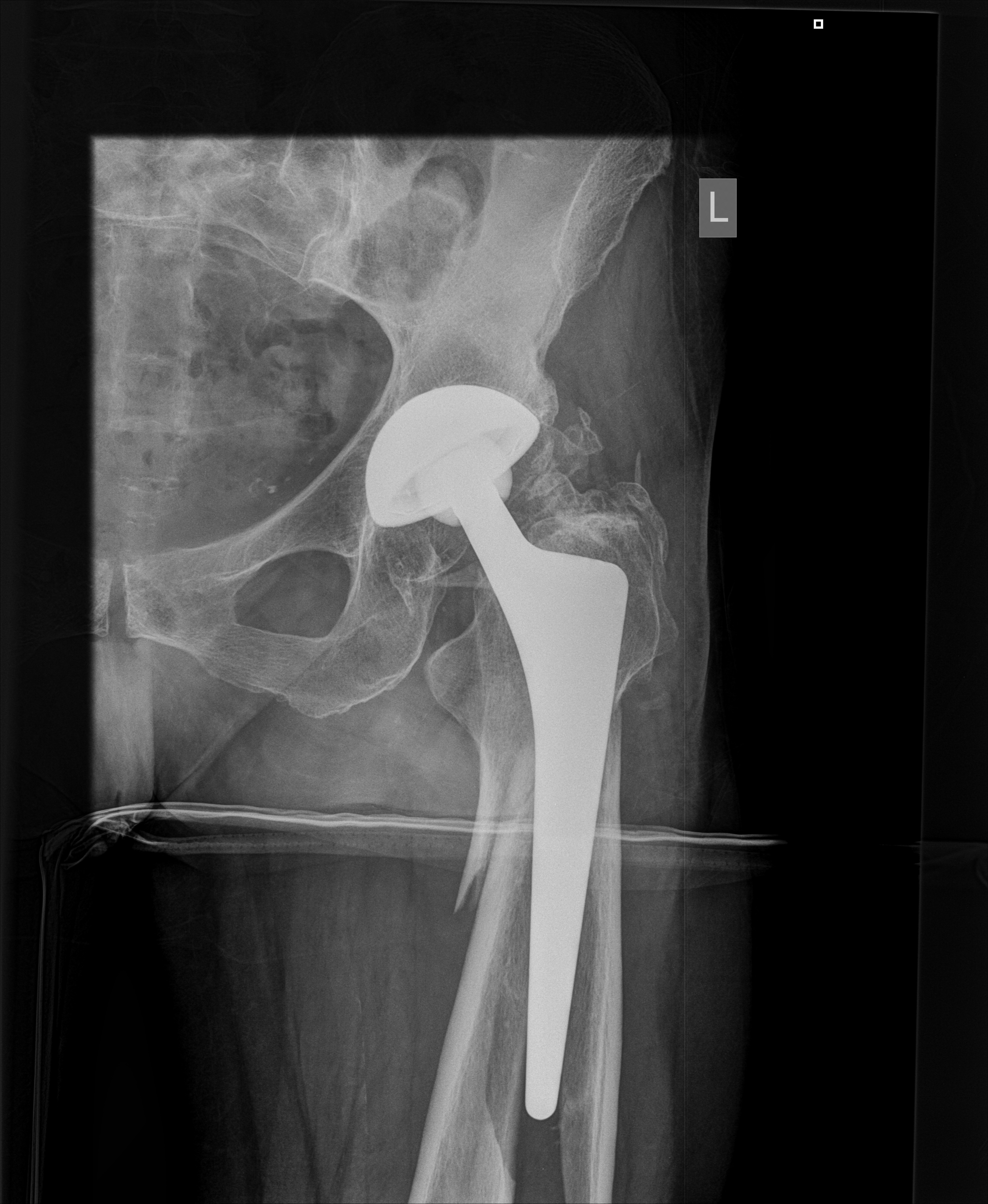

Supplement: sj-jpg-6-hpi-10.1177_11207000211017115 – Supplemental material for Treatment of periprosthetic femoral fractures following total hip arthroplasty: results of an online survey of the European Hip Society [file sj-jpg-6-hpi-10.1177_11207000211017115.jpg]

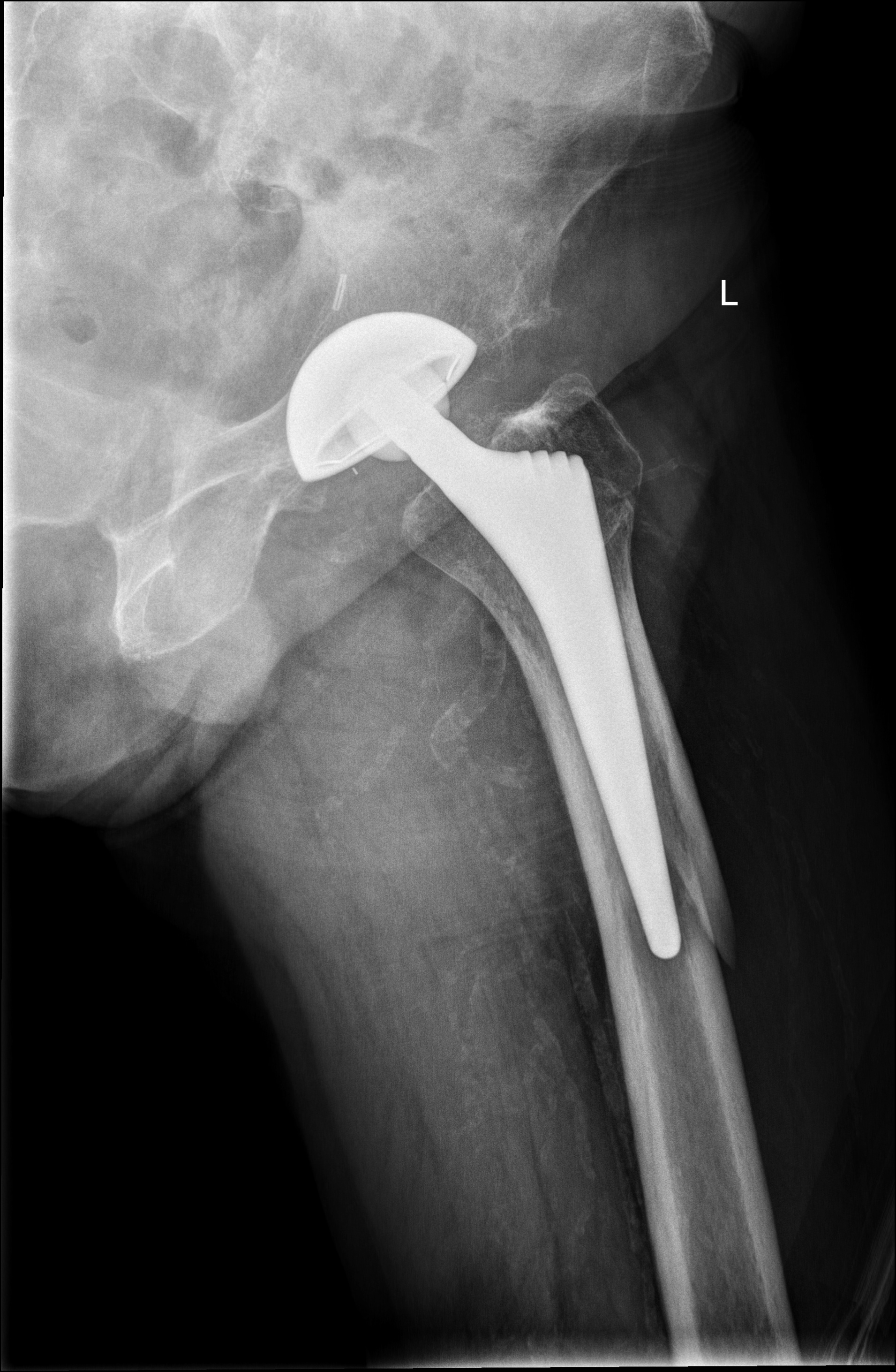

Supplement: sj-jpg-7-hpi-10.1177_11207000211017115 – Supplemental material for Treatment of periprosthetic femoral fractures following total hip arthroplasty: results of an online survey of the European Hip Society [file sj-jpg-7-hpi-10.1177_11207000211017115.jpg]

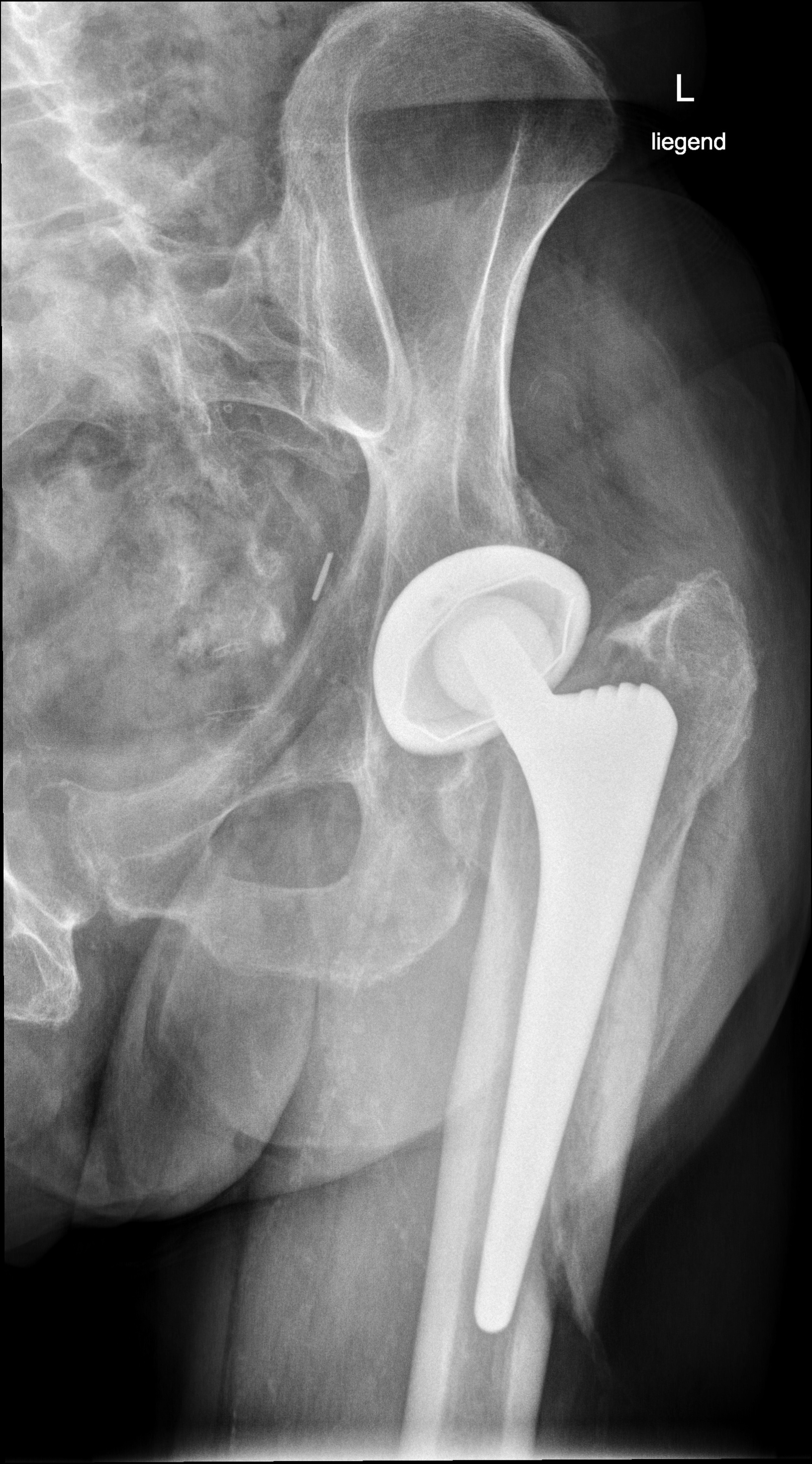

Supplement: sj-jpg-8-hpi-10.1177_11207000211017115 – Supplemental material for Treatment of periprosthetic femoral fractures following total hip arthroplasty: results of an online survey of the European Hip Society [file sj-jpg-8-hpi-10.1177_11207000211017115.jpg]
